# Knee Dislocation With Vascular and Nerve Injury in a Professional Football Player: Return to Play

**DOI:** 10.7759/cureus.21607

**Published:** 2022-01-25

**Authors:** Bernardo Moreno, Pedro Vaz, Bruna Melo, Mafalda Cunha, Rui Vaz

**Affiliations:** 1 Physical Medicine and Rehabilitation, Hospital Senhora da Oliveira - Guimarães, Guimarães, PRT; 2 Orthopaedics and Traumatology, Centro Hospitalar de Entre o Douro e Vouga, Santa Maria da Feira, PRT

**Keywords:** knee injuries, multi-ligamentar knee tear, sports injury, popliteal artery, traumatic knee dislocation

## Abstract

Traumatic knee dislocations are serious and complex injuries, defined as clinical and/or radiological loss of tibiofemoral congruence, which may represent real emergencies to the integrity of the affected limb. This lesion is responsible for multi-ligament tears but the most serious possible complications are related to vascular and peripheral nerve injuries.

Recent studies show that surgical treatment has better functional results and higher return rates to sports practice compared to conservative treatment. However, there is still no consensus on the ideal surgical technique and the timing of surgery. After conservative management or surgical treatment, rehabilitation treatment plays a key role in the recovery process. There are few studies evaluating the return to competition after traumatic knee dislocation and those athletes who return have difficulty reaching the pre-injury level.

Here, we report the case of a professional football player who suffered a traumatic knee dislocation, with multiple ligament tears associated with vascular and neurological damage. Three hours after the initial lesion a double interposition bypass was done with the great saphenous vein, returning flow distally. It was decided not to perform ligament surgery. Two years after a long and intense rehabilitation program the athlete successfully returned to competition.

## Introduction

Traumatic knee dislocations are serious and complex injuries, defined as clinical and/or radiological loss of tibiofemoral congruence, which may represent real emergencies to the integrity of the affected limb. They are relatively uncommon, with an estimated incidence of 29.12 knee dislocations per one million person-years [1], constituting less than 0.02% of all orthopedic injuries in the general population [2] and less than 0.5% of all dislocations joints [3]. 33% of these injuries occur in activities related to sports and 50% occur in car accidents.

In sports, almost 67% of the injured athletes are male, half of them aged between 15-29 years and its highest incidence is associated with extreme adventure sports [1]. In football, there are not enough studies that report the incidence rate of knee dislocations. The injury mechanism during sports practice can be explained by hyperextension of the knee, or by a direct trauma in the tibia, in an anteroposterior direction, causing a posterior translation of this bone relative to the femur. In cases of an unreduced knee, the deformity is obvious, however up to 50% of knee dislocation cases can be caused by spontaneous reduction prior to clinical evaluation, and in some cases, it can be more difficult to diagnose. In these cases, a high degree of suspicion should be maintained to aid diagnosis and exclude associated lesions [4].

Two classification systems have been used to describe knee dislocations, the Kennedy classification system and the Schenck system. Regarding the first one, this classification system stratifies the lesion based on its mechanism of injury: the direction of translation of the tibia relative to the femur. The main disadvantages of this system are: the injured structures are only presumed based on the lesion mechanism and many possible combinations of ligament tears are not well defined. Furthermore, when spontaneous reduction occurs, it is not possible to classify [5]. Concerning the Schenck system, it classifies knee dislocations based on the affected ligament structures. Due to its greater precision, this classification system has more advantages in the decision to treat and in assessing the severity of the injury [6].

The knee dislocations are responsible for multi-ligament tears and can also be the cause of fractures (including avulsion) of the distal femur and proximal tíbia, meniscal and articular cartilage injuries, associated with worse functional outcomes [7]. Despite the seriousness of the involvement of these structures, the most serious possible complications are related to vascular and peripheral nerve injuries.

Up to 64% of knee dislocation cases can be associated with vascular injuries [8], most frequently affecting the popliteal artery (50% of these cases) [7], and a higher risk of vascular injury is associated with posterior knee dislocations, open dislocations and Knee Dislocation (KD) III lateral (L) lesion (injury with tears of anterior cruciate ligament, posterior cruciate ligament and lateral collateral ligament classified in Schenck classification system) [9]. In sports, knee dislocations are associated with a lower rate of vascular injury compared to car accidents injuries [10]. Early detection and treatment of vascular injury are crucial and the amputation rate with early intervention (within a period of less than six to eight hours after injury) is around 11% [11], compared to amputation rates of 86%, if the arterial flow is restored later than that period [7].

Peripheral nerve injuries represent one of the worst complications resulting from traumatic knee dislocations. The common peroneal nerve is the most affected nerve, with an average of 33% of the cases and more than half of these patients do not recover from the neurological deficit [9]. Treatment options for more severe injuries include supportive products for ankle and foot stabilization, neurolysis, tendon transfer, nerve transfer, or combined nerve/tendon transfer [7].

The permanent treatment of ligament injuries may vary from conservative management with prolonged immobilization of the limb to allow the healing of the articular capsule and collateral ligaments, to surgical treatment with affected structures repair or reconstruction [12]. Recent studies show that surgical treatment has better functional results and higher return rates to sports practice compared to conservative treatment. However there is still no consensus on the ideal surgical technique and with the timing of surgery - some studies advocate late surgical treatment (at least three weeks after the injury), while others recommend early surgical reconstruction, within an interval shorter than three weeks [4].

After conservative management or surgical treatment, rehabilitation treatment will play a key role in the recovery process. This long process can last at least nine to 12 months and will depend on the injury of the different structures, the treatment performed (conservative or surgical), and the ligament instability. The intention is functional mobility recovery, joint stability restoration, muscle strengthening, and proprioceptive training [13]. After this injury and initial treatment, in the medium and long term, the most common complications are persistent pain, arthrofibrosis, and persistent instability.

Approximately 25-68% of patients will register chronic pain of variable intensity and multifactorial causes. Between 5-71% of patients develop arthrofibrosis, with a higher risk for athletes who were treated with early simultaneous repair/reconstruction of three or more ligaments, and in 29% of these cases, it is necessary to perform a new surgery for lysis of adhesions. The prevalence of persistent instability in at least one plane, even after surgery, is 42% [14].

There are few studies evaluating the return to competition after traumatic knee dislocation but in one of them, Hirschmann et al. evaluated 26 elite athletes with traumatic knee dislocations and of the 24 selected athletes, 19 returned with some level of sports participation, although only eight reached the pre-injury level [15]. No studies of knee dislocation and associated vascular injury were found.

Our case reports the injury and treatment of a professional football player who suffered a traumatic knee dislocation with associated vascular and neurological damage and the subsequent return to sports practice.

## Case presentation

**Figure 1:**
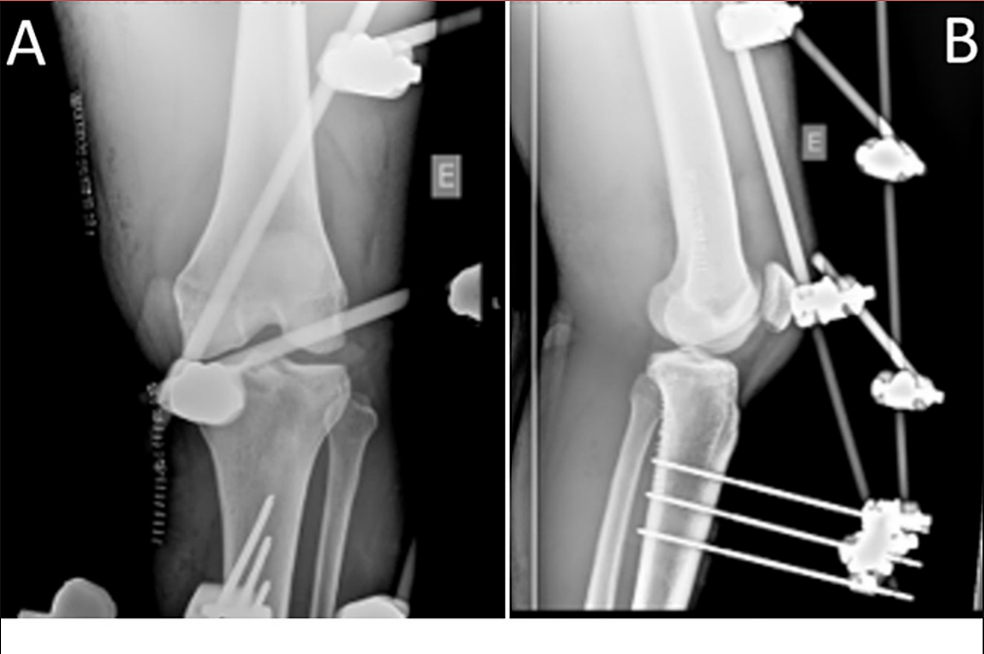
Radiograph of knee joint after external fixator application. A – posteroanterior view; B- lateral view

A 21-year-old professional football player was in the middle of a football match, when he planted his left foot on the field, with his left knee in extension, he suffered a direct trauma to the anterior surface of the tibia, with an anteroposterior direction, resulting in knee deformity. The athlete's lower limb was promptly immobilized and he was transferred to the nearest hospital. In the hospital emergency room, 30 minutes after the trauma, knee radiography was performed, with the confirmation of knee posterior dislocation. The dislocation was promptly reduced and immobilized with a positioning splint. Despite the correct reduction, the athlete's foot was pale, with the absence of capillary filling and ipsilateral distal arterial pulses. Doppler flow was also not identified. The patient was promptly transferred to a nearby Central Hospital for evaluation by Vascular Surgery. Three hours after the initial injury, the athlete was taken to the Central Hospital operating room for angiography and possible arterial repair. Intraoperatively, acute occlusion of the popliteal artery was observed at the first and second portions, with the permeability of the third portion. A double interposition bypass was done with the great saphenous vein, returning flow distally, despite the severity of the arterial injury. At the same surgical time, an orthopedic team applied an external fixator to immobilize and stabilize the knee (Figure 1).

The athlete returned to the initial hospital two days after the injury. During the physical examination, the dorsalis pedis and posterior tibial pulses were present. He had hypoesthesia in common peroneal nerve distribution and decreased muscle strength (grade 1 on the Medical Research Council Scale) in the ankle dorsiflexors and evertors.

Six weeks after the initial injury, a surgery was performed to extract the external fixators from the tibia and femur and, with the patient sedated, due to joint stiffness, the knee was manipulated to obtain a passive range of motion between 0-90º. The use of a knee joint immobilizing splint (DePuy type) and an anti-equine splint on the ankle was indicated.

Eight weeks after the initial injury, an MRI revealed a KD III medial (M) injury, with complete anterior cruciate ligament (ACL) tear; partial posterior cruciate ligament (PCL) tear; partial tear of the medial collateral ligament (MCL), and popliteal tendon; and subchondral fracture at the anterolateral region. The presence of moderate intra-articular effusion was also evident.

In the same week, due to suspicion of peripheral nerve injury, an electromyographic study was performed. A severe incomplete axonal lesion of the common peroneal nerve was evidenced, with severe denervation and still without effective reinnervation of the dependent muscles. The tibial nerve presented moderate injury, with active denervation criteria, but already with signs of dependent muscles effective reinnervation.

At this stage, considering the previous arterial injury and intra-articular inflammation, it was decided not to perform ligament surgery. Therefore, he started a rehabilitation program, initially to control pain and local inflammation and to work on the improvement of knee joint active range of motion. Neuromuscular stimulation of the denervated musculature and of the knee flexors and extensors was also performed. After improving the knee joint function and the function of the common peroneal and tibial nerves, both at the sensory and motor levels, the athlete progressed in his training, introducing strength training, with a special focus on the knee flexor and extensor muscles and on proprioceptive training. During follow-up, quadriceps and hamstring and ankle dorsiflexion and eversion strength improved, and high-intensity training was initiated.

The electromyography study was repeated nine months after the initial injury, revealing the presence of severe and incomplete axonal injury of the left common peroneal nerve, but with signs of effective reinnervation in progress and with normalization of the electromyographic values ​​of the tibial nerve.

Sixteen months after the injury, he still had a slight decrease in the quadriceps, hamstrings, and tibiotarsal dorsiflexion strength, the knee was stable in the varus and valgus stress test, but unstable in the evaluation of instability in anterior/posterior translation. The rehabilitation process was continued.

Despite the risks and joint instability, two years after the initial injury, the athlete, already with full recovery from the common peroneal nerve injury, as well as preserved muscle strength compared to the contralateral side, returned to the competition using a knee joint brace to prevent mediolateral instability and to prevent knee joint hyperextension mechanisms.

## Discussion

Traumatic knee dislocations are considered one of the most challenging injuries, both for the athlete and for the medical team, compromising the possibility of returning to competition and even threatening the limb's viability [15]. Knee dislocations must be reduced and stabilized as quickly as possible, with imaging confirmation. As many of these lesions reduce spontaneously, even without evident deformity, a careful, complete, and detailed evaluation must be carried out, with special attention to vascular and neurological alterations, with the emergent treatment of any vascular compromise, if present [2].

An arterial Doppler ultrasound and/or an ankle-brachial index test should always be performed and in case of doubt (even if minimal), arteriography should still be done. Clarifying the mechanism of injury may also be useful, once anterior and posterior knee dislocations are associated with a concomitant vascular injury in approximately 40% and 50%, respectively.

The permanent treatment of acute knee dislocations remains a case of debate and in recent years, it has been shown that ligament reconstruction, especially in cases of patients who practice sports with multi-ligament injury, is associated with better functional and joint mobility results; and athletes undergoing surgery up to three weeks after the injury have been associated with a higher rate of return to sports activity. Conservative management with a joint splint is often chosen if the joint appears relatively stable after reduction. It is also considered the best treatment option for older, sedentary patients with mediolateral stability or preserved collateral ligaments [7].

This clinical report is one of the few in the literature about an athlete with a traumatic knee dislocation, associated with vascular and neurological injury, who returned to competition, with conservative treatment.

In theory, a young athlete with a knee dislocation classified as KD III M vascular injury (C) nerve injury (N) (Table 1) should receive surgical treatment with ligament reconstruction, however, considering the severity of the surgically corrected vascular lesion and the knee stiffness, it was decided not to perform surgical treatment due to the risk of losing the corrected vascular viability and leading to the formation of scar tissue, that could worsen the joint movement capacity, in particularly the knee extension [4]. Therefore, the conservative management was decided with the objective of preserving the knee integrity and avoiding the risk of losing limb functionality for activities of daily living.

**Table 1:** Schenck Classification System ACL: anterior cruciate ligament; C: Vascular injury; KD: Knee dislocation; L: Lateral; LCL: Lateral collateral ligament; M: Medial; MCL: Medial Collateral Ligament; N: Nerve injury; PCL: Posterior cruciate ligament [6].

Category	Description
KD I	ACL or PCL injury
KD II	ACL and PCL injury
KD III M	ACL, PCL and MCL injury
KD III L	ACL, PCL and LCL injury
KD IV	ACL, PCL, LCL and MCL injury
KD V	Multiligamentous injury with peri-articular fracture
Letter annotation added to category with repective injury present: N, C	

Through the rehabilitation treatment with the recovery of joint range of motion, muscle strength, and proprioceptive training, the athlete showed favorable evolution, so during this process, it was decided to readjust the rehabilitation treatment goal to return to sports competition.

Another important aspect of this case was the common peroneal and tibial nerves injury, which the recovery was facilitated with the treatment applied to maintain and stimulate the dependent muscles trophism and preventing tendon shortening and consequent equinus of the tibiotarsal joint.

## Conclusions

This case highlights the importance of a careful examination of patients with knee dislocation, even after knee reduction. If the popliteal artery disruption was initially missed or if the arterial flow was restored later, it would be possible that the young athlete could have lost his lower leg. Mandatory vascular evaluations with arterial Doppler ultrasound and/or an ankle-brachial index test should always be performed and in case of doubt (even if minimal), arteriography should be considered. After emergent treatment and stabilization of any vascular compromise, if present, the approach to ligament reconstruction is largely based on surgical treatment. However, each case must be evaluated individually.

In this case, despite having undergone conservative treatment, the athlete managed to return to professional sports activity. Despite being a relatively uncommon injury, more studies are needed in order to review the treatment algorithms, to reduce the morbidity and sequelae of these injuries, and to facilitate the athletes' return to play.
